# Impact of the Cathode Pt Loading on PEMFC Contamination by Several Airborne Contaminants

**DOI:** 10.3390/molecules25051060

**Published:** 2020-02-27

**Authors:** Jean St-Pierre, Yunfeng Zhai

**Affiliations:** Hawaii Natural Energy Institute, University of Hawaii—Manoa, Honolulu, HI 96822, USA; yunfeng@hawaii.edu

**Keywords:** proton exchange membrane fuel cells, durability, contamination, cathode, catalyst loading

## Abstract

Proton exchange membrane fuel cells (PEMFCs) with 0.1 and 0.4 mg Pt cm^−2^ cathode catalyst loadings were separately contaminated with seven organic species: Acetonitrile, acetylene, bromomethane, iso-propanol, methyl methacrylate, naphthalene, and propene. The lower catalyst loading led to larger cell voltage losses at the steady state. Three closely related electrical equivalent circuits were used to fit impedance spectra obtained before, during, and after contamination, which revealed that the cell voltage loss was due to higher kinetic and mass transfer resistances. A significant correlation was not found between the steady-state cell voltage loss and the sum of the kinetic and mass transfer resistance changes. Major increases in research program costs and efforts would be required to find a predictive correlation, which suggests a focus on contamination prevention and recovery measures rather than contamination mechanisms.

## 1. Introduction

Vehicles propelled by proton exchange membrane fuel cells (PEMFCs) are already commercially available. However, opportunities still exist to improve the technology because it is not expected to mature within the foreseeable future [1]. For instance, research activities are still ongoing to reduce cost while maintaining durability with a lower amount of Pt catalyst [2]. Contaminants in air jeopardize PEMFC operation by increasing the cell voltage degradation rate [3] if the intake filter [4] is saturated or damaged. Therefore, risks associated with contamination of low Pt loaded PEMFCs need to be assessed to support commercialization. Furthermore, fuel cell design robustness could be improved by integrating additional mitigation approaches derived from contamination mechanisms.

Only a few publications discuss the impact of the anode catalyst loading during PEMFC exposures to reformate fuel contaminants, such as CO, CO_2_, H_2_S, NH_3,_ and halogenated compounds. All of these species are included in the hydrogen fuel standard [5]. For CO and H_2_S, a lower Pt or PtRu catalyst loading generally leads to an increase in the anode overpotential [6,7,8,9,10]. However, it was reported that for H_2_S, the catalyst loading effect disappears for values equal to or below 25 μg cm^−2^ [10]. An effect was not observed with the weak contaminant CO_2_, which is attributed to a concentration that was substantially lower (1%) [7] than in a typical reformate (10–20%) [11]. The same situation was noted for NH_3_, which is assigned to a rapid conversion to NH_4_^+^ in the presence of protons or water [12,13], followed by ion exchange with ionomer H^+^ and transport to the cathode away from the anode under the influence of the electric field [14,15]. For halogenated compounds, a decrease in the Pt catalyst loading of both electrodes led to a faster degradation rate in the presence of HCl in both reactant stream humidifiers [16]. The effect of the anode Pt catalyst loading was exploited to develop sensor cells that are more sensitive to contamination by CO, H_2_S, and NH_3_ in H_2_. These sensors were either based on a PEMFC [17] or a H_2_ pump [18] design. Only two PEMFC contamination documents focusing on the cathode catalyst loading effect were found [19,20]. However, contamination data in [19] are not directly comparable because both the catalyst layer design and catalyst loading were concurrently altered. The authors also refer to 10 ppb SO_2_ data obtained by another group that showed more severe fuel cell damage with a catalyst loading decrease from 0.4 to 0.3 mg Pt cm^−2^. In contrast, the effect of 2,6-diaminotoluene, a species that leaches out of the fuel cell system balance of plant materials, was more impactful after the Pt catalyst loading was lowered from 0.4 to 0.1 mg Pt cm^−2^ [20]. In comparison to the anode, the higher cathode potential is expected to affect the contamination mechanism with, for example, a different Pt surface charge, altered contaminant adsorbates and reaction intermediates, catalyst coverage, and cell voltage loss. This situation is exacerbated with a catalyst loading change, which affects the overpotential of the irreversible oxygen reduction reaction and the cathode potential. Information about chemical and electrochemical reactions for specific contaminants may be available in the literature. However, the presence of relevant cathode reactants, oxygen and water, may not be considered. For instance, novel intermediates or products were not detected with chlorobenzene in air [21]. However, the presence of acetylene in air led to small amounts of methane [22] that were not expected based on acetylene chemistry and electrochemistry. Therefore, tests completed under these significantly different operating conditions are needed in part because contaminant reactions are not currently predictable in assessing catalyst coverage and cell voltage loss.

This report documents the impact of the cathode Pt catalyst loading effect for PEMFCs contaminated with seven model organic airborne species, which were previously evaluated and selected from a larger pool of 21 contaminants [23]: Acetonitrile (nitrile), acetylene (alkyne), bromomethane (halocarbon), iso-propanol (alcohol), methyl methacrylate (ester), naphthalene (polycyclic aromatic), and propene (alkene). Cell voltage transients obtained under galvanostatic conditions were recorded for this analysis. Additionally, impedance spectroscopy data were acquired to facilitate the development of predictive correlations and contamination mechanisms.

## 2. Results and Discussion

### 2.1. Cell Voltage Transients

**Figure 1:**
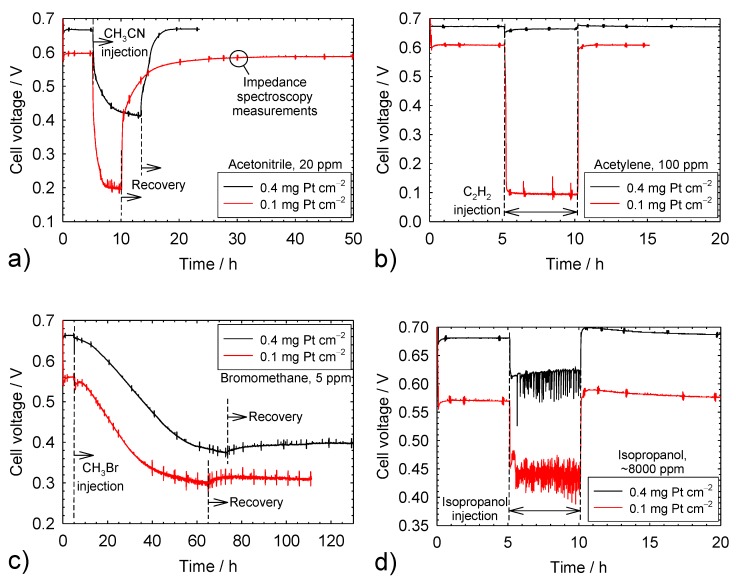

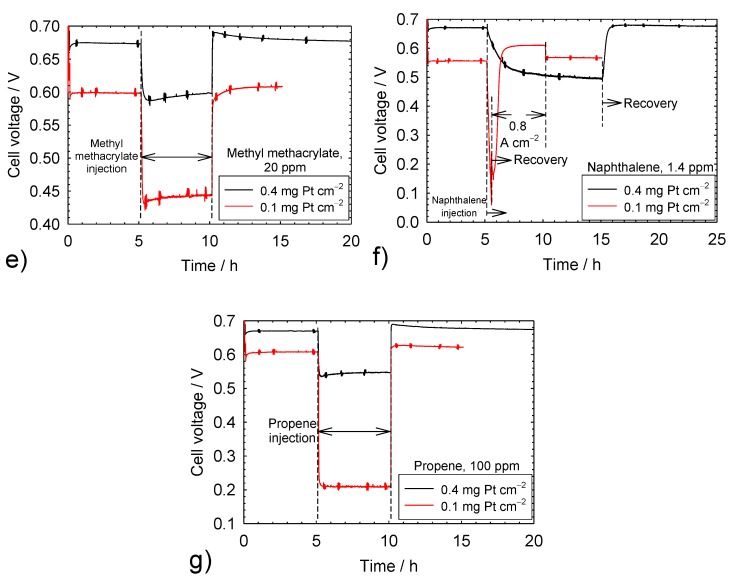
Cell voltage transients resulting from a temporary contaminant injection. (**a**) Acetonitrile; (**b**) acetylene; (**c**) bromomethane; (**d**) isopropanol; (**e**) methyl methacrylate; (**f**) naphthalene; (**g**) propene.

Figure 1a depicts voltage transients for cells temporarily exposed to 20 ppm CH_3_CN. The cell voltage for the first 5 h is constant and higher for the 0.4 mg Pt cm^−2^ catalyst loading. This observation is consistent with previously published data for Gore catalyst coated membranes with the same cathode catalyst loadings and gas diffusion layers (Sigracet 25 BC) [24]. After approximately 5 h of operation, acetonitrile was injected into the cell, which led to a rapid cell voltage decrease that progressively slowed until a steady state was reached. For acetonitrile, the cell voltage loss was larger for the 0.1 mg Pt cm^−2^ catalyst loading. Subsequently, the acetonitrile injection was interrupted, which quickly initiated a voltage recovery that gradually decelerated until a new steady state was reached. For acetonitrile, the cell voltage after recovery coincided with the value before contaminant injection. Acetonitrile contamination and recovery transients are qualitatively and quantitatively consistent with prior results [23,25,26,27,28]. At irregular intervals and during all baseline, contamination, and recovery stages, cell voltage transients were minimally disrupted for a short period by impedance spectroscopy measurements and the superimposition of a current signal of a small amplitude and variable frequency.

Figure 1b–g illustrates voltage transients for the other contaminants. Most of these transients share common features, including a similar initial baseline voltage, a relatively rapid voltage decrease until a steady state is reached, and a complete voltage recovery after contaminant injection was stopped. However, bromomethane transients were significantly slower and only a small fraction of the voltage loss was recovered (Figure 1c). This behavior is the result of a rapid bromomethane hydrolysis within the cell, producing methanol and bromide [28,29,30]. The effective bromomethane concentration is lower than the nominal value, whereas bromide is prevented from penetrating the ionomer by Donnan exclusion [12]. This situation delays the stronger and inhibiting adsorption of bromomethane and bromide on the catalyst surface. The removal of bromide from the catalyst surface is equally hindered due to an unfavorable cathode potential that is significantly higher than the potential of zero charge, preventing bromide desorption and Donnan exclusion, which explains the incomplete voltage recovery. During isopropanol contamination, the voltage is characterized by rapid fluctuations (Figure 1d), which were not observed for lower isopropanol concentrations [23,31]. These fluctuations are attributed to isopropanol, a surfactant commonly used to disperse Pt/C catalyst particles in solution [32], which adsorbs on carbon materials (gas diffusion layer, catalyst support) [33] and modifies liquid water management (buildup and release of liquid water drops), as previously proposed for acetylene [34]. A higher number of buildup and release events of water drops and a higher voltage fluctuation frequency for the lower catalyst loading (Figure 1d) may be related to the lower cathode potential (cell voltage compensated by a similar ohmic drop), which leaves a higher proportion of isopropanol surfactant unoxidized (oxidation initiated at a potential above 0.32 V vs. the reversible hydrogen electrode (RHE) [35]) and more hydrophilic carbon surfaces. The effect of naphthalene was rapid and severe for the 0.1 mg Pt cm^−2^ catalyst loading (Figure 1f). As a result, the current density was temporarily lowered and the contaminant injection was interrupted before a steady state was obtained to avoid an automatic test station shutdown. Contamination and recovery transients are qualitatively and quantitatively consistent with the prior results for acetylene [23,36,37,38,39], bromomethane [23,28,29,30], isopropanol [23,31], methyl methacrylate [23,31], naphthalene [23,40], and propene [23,28,31,41].

Table 1 summarizes steady-state cell voltages before, during, and after contamination as well as the cell voltage change during and after contamination for both catalyst loadings. The cell voltage decrease during the contamination period is generally higher for the 0.1 mg Pt cm^−2^ catalyst loading (23% to 89% in comparison to 1.2% to 43%). After the recovery period, the cell voltage change is minimal and independent of the catalyst loading, varying from −1.7% to 2%, with the exception of bromomethane (−40% to −45%). The larger cell voltage loss during contamination for the low catalyst loading is an important consideration for the selection of tolerance limits for commercially relevant catalyst loadings. Data obtained with a 0.4 mg Pt cm^−2^ catalyst loading were used to derive tolerance limits [42]. The data of Table 1 suggest that these tolerance limits require a revision for a 0.1 mg Pt cm^−2^ catalyst loading and additional tests carried out over a range of concentrations. In contrast, International Organization for Standardization (ISO) tolerance limits for hydrogen contaminants [5,43], which do not take account of the catalyst loading effect, were deemed too strict for formaldehyde and formic acid, a low anode catalyst loading of 0.05 mg Pt cm^−2^, and automotive operating conditions (high fuel utilization, fuel recirculation) [44]. The formaldehyde tolerance limit was recently modified from 10 [43] to 200 ppb [5].

The magnitude of the cell voltage change during contamination (Table 1) with catalyst loading is species-dependent. For instance, the catalyst loading hardly affected the cell voltage loss for bromomethane (−43% and −47%), whereas for acetylene, the cell voltage loss substantially increased from −1.2% to −85% with a catalyst loading decrease. This observation is attributed to different contamination mechanisms. Impedance spectroscopy data obtained during contamination by all Table 1 species and with a 0.4 mg Pt cm^−2^ catalyst loading revealed that kinetic, ohmic, and mass transport overpotentials were impacted [42]. These and additional impedance spectroscopy data acquired with a 0.1 mg Pt cm^−2^ catalyst loading were analyzed to evaluate the existence of a correlation between these resistances and the cell voltage loss due to contamination at the steady state.

### 2.2. Impedance Spectra

**Figure 2:**
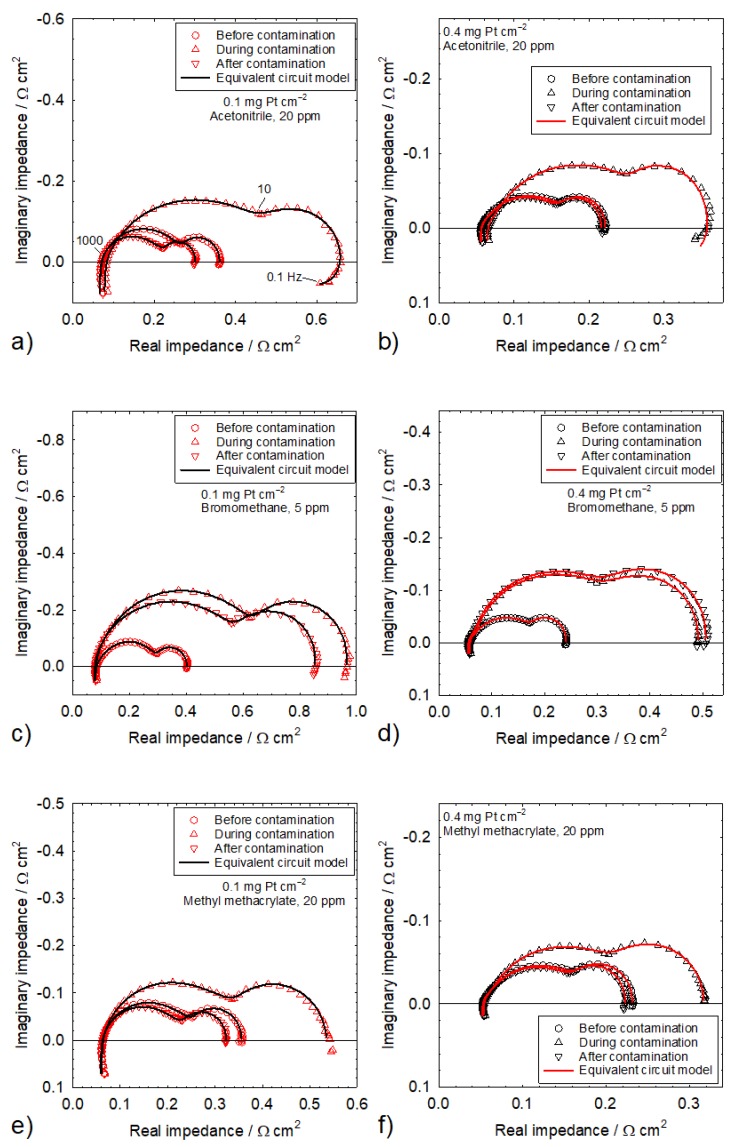
Impedance spectra before, during, and after contamination by acetonitrile in (**a**) and (**b**), bromomethane in (**c**) and (**d**), and methyl methacrylate in (**e**) and (**f**) for Pt catalyst loadings of 0.1 mg cm^−2^ in (**a**), (**c**), and (**e**), and 0.4 mg cm^−2^ in (**b**), (**d**), and (**f**).

**Figure 3:**
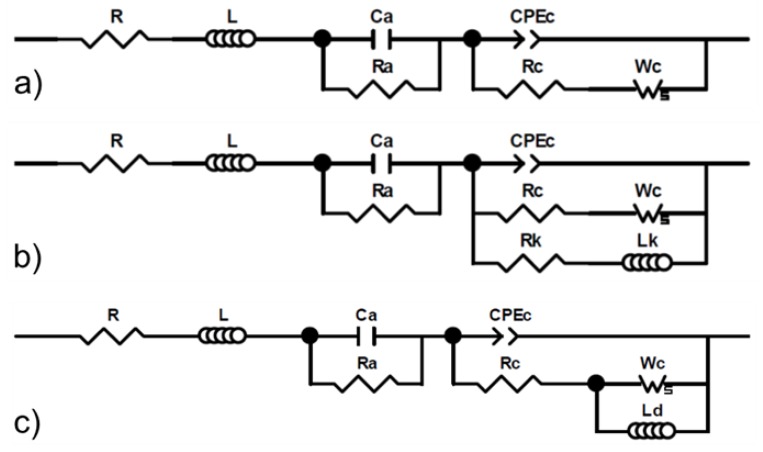
Equivalent circuit models for a proton exchange membrane fuel cell (PEMFC). (**a**) The model previously derived for SO_2_ contamination and used for all 7 organic contaminants investigated in this work; (**b**) the model previously derived to capture low-frequency inductive data in the absence of contaminants and used to fit data during acetonitrile contamination (0.1 mg Pt cm^−2^); (**c**) the modified model (**b**) to capture low-frequency inductive data obtained during acetonitrile (0.4 mg Pt cm^−2^) and propene (0.1 mg Pt cm^−2^) contamination.

Figure 2a shows impedance spectra (Nyquist representation) for a 0.1 mg Pt cm^−2^ catalyst loading, before, during, and after acetonitrile contamination. All three spectra share the same features and have two main loops that are respectively attributed to oxygen reduction (medium frequencies) and oxygen mass transfer (low frequencies) [45]. A third loop ascribed to hydrogen oxidation is barely visible as a hump at high frequencies [45]. The high-frequency intercept represents the ohmic resistance, which is mostly caused by the poorly conducting membrane [45]. Multiple explanations were proposed for the inductive impedance values at the lowest and highest frequencies, including electrical cables [46,47] for high frequencies, and processes involving side reactions with intermediate species [47], oxide growth [48], or a slow ionomer water uptake/release [49] for low frequencies. Most of these considerations were either ignored because they did not focus on relevant aspects (electrical cables) or were easily dismissed because, in the absence of contaminants, the cathode potential was too low for Pt oxidation and the sub-saturated air stream did not yield an inductive behavior. For the 0.4 mg Pt cm^−2^ catalyst loading, the average cell voltage of 0.671 V (Table 1) compensated with an ohmic loss of 0.1 V for a worst-case scenario (1 A cm^−2^ × 0.1 Ω cm^2^ from the high-frequency intercepts in Figure 2a) leads to a cathode potential of 0.771 V vs. RHE, which is lower than the smallest Pt oxidation potential of 0.837 V vs. RHE [50]. Acetonitrile contamination causes an increase in the high-frequency intercept and a diameter increase for both main loops (Figure 2a). An increase in ohmic loss was only observed with acetonitrile, owing to the production of ammonium cations by hydrolysis, which displace protons as the main charge carriers in the ionomer [28,51]. In relative terms, this effect is significantly smaller than the kinetic and mass transfer effects, with an approximate doubling of both oxygen reduction and transport loop diameters. However, because the effect is cumulative, a larger change is observed for a longer exposure duration [26]. After contamination, the high-frequency intercept returns to its original value, and both main loops decrease in size to a diameter that is smaller than the original value. These impedance spectra agree with prior results [25,26,27,28]. However, smaller kinetic and mass transfer loops are inconsistent with a complete cell voltage recovery (Figure 1a, Table 1). This observation is possibly due to subtle structural or other changes that are not detectable by cell voltage measurements, such as Pt surface reconstruction in the presence of foreign species [52,53]. The oxygen reduction and mass transfer resistances before, during, and after contamination were generally obtained by curve-fitting an equivalent circuit developed for a PEMFC contaminated with SO_2_ (Figure 3a) [54]. Resistances during contamination for acetonitrile and a 0.1 mg Pt cm^−2^ catalyst loading were derived using a modified equivalent circuit that accounts for the inductive behavior at low frequencies (Figure 3b) [55,56]. Resistances during contamination for acetonitrile (0.4 mg Pt cm^−2^) and propene (0.1 mg Pt cm^−2^) were obtained using a modified version of the Figure 3b equivalent circuit by omitting the cathode resistance Rk (Figure 3b) to limit the number of parameters (Figure 3c). The impedance spectra are accurately represented by the equivalent circuit models (Figure 2a–f). The resistance values are discussed later in this section.

Most of the other impedance spectra for both catalyst loadings and all contaminants are equally well represented by the equivalent circuits shown in Figure 3a,c. For this reason, only a selection is given in Figure 2. A few spectra could not be fitted with any of the equivalent circuits in Figure 3a–c for a few 0.1 mg Pt cm^−2^ catalyst loading cases. For acetylene, the impedance spectrum during contamination was approximately a single loop of a large diameter that could not be fitted to a two-loop equivalent circuit. For isopropanol, cell voltage fluctuations during contamination (Figure 1d) created a low frequency artefact that also prevented the use of the equivalent circuits of Figure 3a or Figure 3c. For naphthalene, the cell voltage transient was interrupted before a steady state was obtained (Figure 1f), which also led to a low-frequency artefact that could not be fitted to the equivalent circuits of Figure 3a–c. The impedance spectra agree with the prior results for acetonitrile [25,26,27,28], acetylene [36,37,38], bromomethane [28,29,30], isopropanol [31], methyl methacrylate [31], naphthalene [40], and propene [28,31,41].

**Figure 4:**
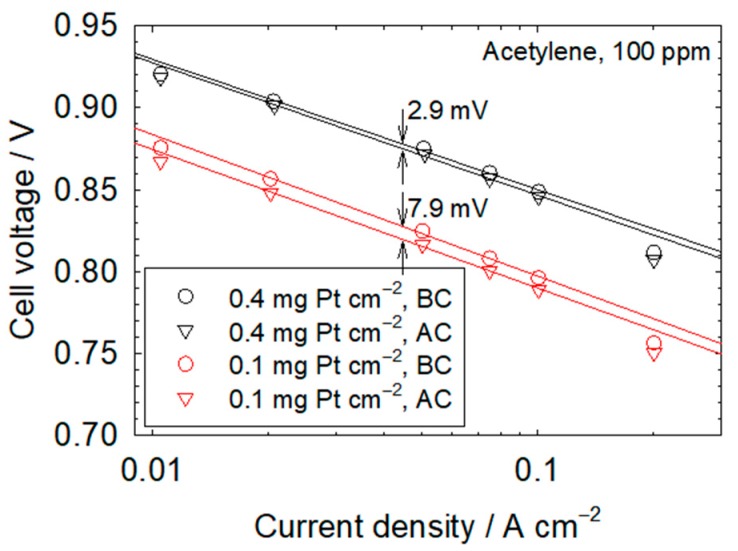
Tafel plots before contamination (BC) and after contamination (AC) with 100 ppm acetylene for 0.1 and 0.4 mg Pt cm^−2^ catalyst loadings. The change in cell voltage between plots at a current density of 0.0447 A cm^−2^, a value located in the middle of the range used to correlate data (0.02 to 0.1 A cm^−2^), ignores the slight change in slope.

Table 2 collects kinetic and mass transfer resistances before, during, and after contamination for both catalyst loadings. Dimensionless kinetic and mass transfer resistances during and after contamination are also given in Table 2. The dimensionless kinetic and mass transfer resistances concurrently increase during contamination and are ≥1.05, with the exception of the 0.93 dimensionless mass transfer resistance for isopropanol and a 0.4 mg Pt cm^−2^ catalyst loading. The isopropanol anomaly may be related to water management, as discussed in the previous section. A hypothesized connection between kinetic and mass transfer resistances during contamination [34] was recently substantiated [57]. Contaminant adsorbates covering the catalyst surface increase the effective current density closer to the limiting value and mass transfer losses in the ionomer layer covering the catalyst. This situation is similar to a decrease in catalyst loading, which has been shown to also increase mass transfer losses [58,59]. The dimensionless kinetic and mass transfer resistances after recovery, with the exception of bromomethane, indicate an incomplete recovery that is less extensive for the lower catalyst loading. For the dimensionless kinetic resistance, values are ≥0.832 and ≥0.95 for, respectively, 0.1 and 0.4 mg Pt cm^−2^ catalyst loadings. For the dimensionless mass transfer resistance, values are ≥0.842 and ≥0.88 for, respectively, 0.1 and 0.4 mg Pt cm^−2^ catalyst loadings. These results are in contrast with the data of Figure 1 and Table 1, showing a complete recovery within experimental error, with the exception of bromomethane. The discrepancy between the recovery extents of cell voltage and kinetic and mass transfer resistances is due to the higher sensitivity of impedance measurements and the movement of the reaction front (current density and catalyst layer effectiveness redistributions over the catalyst layer thickness). The hydrogen peroxide yield is enhanced in the presence of acetonitrile, acetylene, methyl methacrylate, naphthalene, and propene [60,61,62,63]. The elevated level of hydrogen peroxide in turn promotes ionomer degradation [64] and structural modifications to the catalyst layer that are relatively more impactful for the lower catalyst loading. Therefore, in view of the lower cell voltage and cathode potential for a lower catalyst loading (Figure 1, Table 1), a higher hydrogen peroxide yield [60,61,62,63] and ionomer degradation are expected. Tafel plots obtained before and after contamination with acetylene (Figure 4) support this hypothesis, with a larger cell voltage loss for the 0.1 mg Pt cm^−2^ catalyst loading (7.9 mV in comparison to 2.9 mV).

### 2.3. Contaminant Effect Prediction

**Figure 5:**
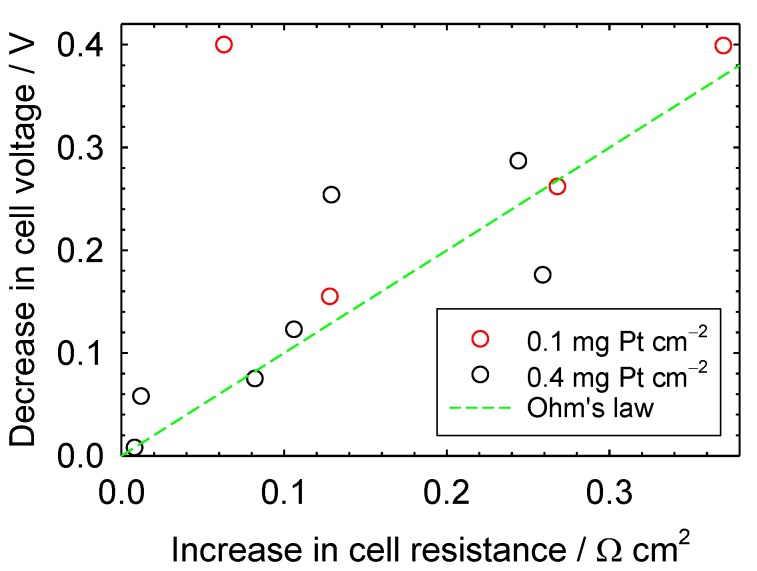
Cell voltage loss as a function of the sum of the changes in kinetic and mass transfer resistance.

The steady-state cell voltage loss during contamination was correlated with the sum of the kinetic and mass transfer resistance changes during contamination (Figure 5). A significant correlation was not identified, as significant deviations from Ohm’s law were noted. Furthermore, it is difficult to argue that there is a catalyst loading effect because the two data sets largely overlap. The absence of a correlation is not surprising, considering the effects of cell design and operating conditions on contamination. Several parameters were mentioned in an earlier attempt to correlate the effect of contaminants on oxygen reduction kinetics [65], including contaminant partial pressure and temperature, exposed Pt surface features (crystal faces, edges), Pt state (reduced or oxidized), phase in contact with the Pt surface (air, ionomer), adsorption isotherms for O_2_, contaminants, and related intermediates and products, and elementary chemical and electrochemical reactions and associated rate constants for O_2_ reduction and contaminant oxidation or reduction. This list is enlarged by factors affecting ohmic and mass transfer losses, including cation and neutral molecules’ absorption isotherms influencing ionomer and membrane ionic conductivity and oxygen permeability by swelling and changing the distance between sulfonate groups, and contaminant scavenging by liquid water modifying the effective contaminant concentration [12,13,66,67,68,69,70,71]. Although cell design and operating conditions were maintained as constant as possible, with the exception of catalyst loading and contaminant concentration, the change in cell resistance is insufficiently precise to capture all contamination nuances and accurately predict the cell voltage loss (Figure 5). An accurate correlation for the cell voltage loss would be useful. However, given the amount of information that will be required and the complexity associated with the derivation of a detailed mathematical model of contamination, a focus on preventive and recovery measures may be more fruitful. This suggestion is reinforced by considering practical aspects, contaminant mixtures [28], and long-term effects [26] that increase the number of contamination parameters and the difficulty in predicting contaminant effects.

## 3. Materials and Methods

A single modified Fuel Cell Technologies cell with an active area of 50 cm^2^ and triple/double serpentine channels for the cathode/anode was used for all experiments. Gore PRIMEA M715 catalyst-coated membranes with a Pt loading of 0.1 or 0.4 mg Pt cm^−2^ (50 % Pt/C) on each side were inserted between SGL Carbon Sigracet 25 BC gas diffusion layers. The cell was operated with a FCATS™ G050 series test station (Green Light Power Technologies). After cell activation, operating conditions were set to air/H_2_, 2/2 stoichiometry, 48.3/48.3 kPa_g_ outlet pressure, 50%/100% relative humidity, 80 °C, and 1 A cm^−2^. Contaminant concentrations varied between 1.4 and ~8000 ppm: Acetonitrile (20 ppm), acetylene (100 ppm), bromomethane (5 ppm), isopropanol (~8000 ppm), methyl methacrylate (20 ppm), naphthalene (1.4 ppm), and propene (100 ppm). Contaminant concentrations were individually and empirically adjusted based on prior experience to cause a perceptible to significant cell voltage decrease at the steady state for the 0.4 mg Pt cm^−2^ catalyst loading, and to leave a sufficient cell voltage window for an additional decrease induced by the lower 0.1 mg Pt cm^−2^ catalyst loading. Contaminants were injected after the air humidifier using air-based gas mixtures for most cases. However, isopropanol and naphthalene were respectively evaporated and sublimated by employing a thermally controlled and calibrated liquid/solid holder. Contaminant injection was initiated after 5 h with an exposure that lasted from less than 1 to ~70 h until a steady state was achieved. After the contamination injection was interrupted, the self-induced recovery was recorded until a steady state was obtained, which necessitated between 5 and ~60 h.

During the galvanostatic experiments, impedance spectra were acquired at irregular intervals by superimposing 0.1 Hz to 10 kHz (10 points per decade) current perturbations that caused a voltage change of ~5 mV. The Solartron SI1260 impedance/gain-phase analyzer was operated with ZPlot^®^ software (Version 2.9c, Scribner Associates, Southern Pines, NC, USA). Measurement accuracy was improved by utilizing Stanford Research SR560 low-noise preamplifiers and by winding up both load-bank cables, which have an equal length, to reduce their inductance. The ZView^®^ software (Version 3.5e, Scribner Associates) was employed for fitting impedance spectra to equivalent circuit models. Polarization curves were only recorded before and after acetylene contamination. Polarization curves were measured by decreasing the current density from 2 to 0 (open circuit voltage) A cm^−2^ in a stepwise fashion, allowing a stabilization time of 15 min at each stage, and otherwise using contamination test operating conditions.

## 4. Conclusions

The effect of Pt catalyst loading on the steady-state cell voltage loss was characterized for seven organic airborne contaminants. Impedance spectroscopy was used to gain mechanistic insight. The steady-state cell voltage loss is mostly attributed to a concurrent increase in both kinetic and mass transfer resistances that is reminiscent of the effect of a decrease in catalyst loading in the absence of a contaminant. Low Pt catalyst loadings generally lead to a larger steady-state cell voltage loss. A significant correlation between the steady-state cell voltage loss and the sum of the kinetic and mass transfer resistance changes was not found, and would only be improved with major increases in cost and effort. For this reason, it is proposed to focus activities on contamination prevention and recovery measures.

For a commercially relevant cathode catalyst loading of 0.1 mg Pt cm^−2^, it would be advantageous to expand the current database to other contaminants and contaminant concentrations for the derivation of tolerance limits to support the design of air filters. Although tolerance limits were previously derived for single contaminants rather than for more practically relevant mixtures [42], for very low contaminant concentrations, tolerance limits may still prove useful because the catalyst surface coverage by contaminant adsorbates may be so small that the different species may not interact. In other words, the effects of all contaminants may be additive. It would also be useful to verify this hypothesis with diluted, single, and multiple contaminant mixtures.

## Figures and Tables

**Table 1:** Steady-state cell voltages at the end of each contamination period, and steady-state cell voltage changes during and after contamination.

Contaminant	Catalyst Loading/mg Pt cm^−2^	Cell Voltage/V			Cell Voltage Percentage Change/%	
		Before Contamination ^1^	During Contamination	After Contamination	During Contamination	After Contamination
Acetonitrile 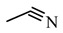	0.1	0.597	0.198	0.587	−67	−1.7
	0.4	0.666	0.412	0.670	−38	0.60
Acetylene 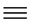	0.1	0.607	0.093	0.608	−85	0.16
	0.4	0.672	0.664	0.672	−1.2	0
Bromomethane 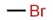	0.1	0.561	0.299	0.309	−47	−45
	0.4	0.663	0.376	0.398	−43	−40
Isopropanol 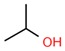	0.1	0.570	0.439	0.577	−23	1.2
	0.4	0.681	0.623	0.687	−8.5	0.88
Methyl methacrylate 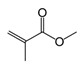	0.1	0.599	0.444	0.608	−26	1.5
	0.4	0.673	0.598	0.678	−11	0.74
Naphthalene 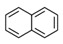	0.1	0.557	0.060 ^2^	0.566	−89	1.6
	0.4	0.671	0.495	0.677	−26	0.89
Propene 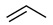	0.1	0.609	0.209	0.621	−66	2.0
	0.4	0.670	0.547	0.674	−18	0.60

^1^ For 0.1 mg Pt cm^−2^, mean = 0.586 V and standard deviation = 0.022 V. For 0.4 mg Pt cm^−2^, mean = 0.671 V and standard deviation = 0.006 V. ^2^ Not at steady state because the cell voltage was still decreasing.

**Table 2:** Steady-state kinetic and mass transfer resistances at the end of each contamination period, and steady-state dimensionless resistances during and after contamination.

Contaminant	Catalyst Loading/mg Pt cm^−2^	Kinetic/Mass Transfer Resistances/Ω cm^2^			Dimensionless Resistance During/After Contamination ^1^	
		Before Contamination	During Contamination	After Contamination	Kinetic	Mass Transfer
Acetonitrile	0.1	0.118/0.095	0.396/0.187	0.108/0.080	3.36/0.915	1.97/0.842
	0.4	0.104/0.056	0.210/0.079	0.099/0.056	2.02/0.952	1.41/1.00
Acetylene	0.1	0.133/0.107	- ^2^	0.117/0.106	-	-
	0.4	0.107/0.061	0.112/0.064	0.109/0.059	1.05/1.02	1.05/0.967
Bromomethane	0.1	0.103/0.109	0.139/0.341	0.285/0.288	1.35/2.77	3.13/2.64
	0.4	0.116/0.062	0.265/0.157	0.274/0.167	2.28/2.36	2.53/2.69
Isopropanol	0.1	0.102/0.100	- ^3^	0.123/0.090	-	-
	0.4	0.100/0.070	0.117/0.065	0.095/0.068	1.17/0.950	0.929/0.971
Methyl methacrylate	0.1	0.121/0.104	0.164/0.189	0.104/0.098	1.36/0.860	1.82/0.942
	0.4	0.111/0.063	0.152/0.104	0.107/0.059	1.37/0.964	1.65/0.937
Naphthalene	0.1	0.115/0.108	- ^3^	0.119/0.097	-	-
	0.4	0.106/0.075	0.288/0.152	0.101/0.066	2.72/0.953	2.03/0.880
Propene	0.1	0.137/0.107	0.189/0.118	0.114/0.107	1.38/0.832	1.10/1.00
	0.4	0.117/0.063	0.152/0.134	- ^4^	1.30/-	2.13/-

^1^ Resistance during/after contamination divided by the resistance before contamination. ^2^ Equivalent circuit models do not fit due to a side surface reaction involving intermediates. ^3^ Artefact created by flooding or rapid change in cell voltage. ^4^ Data was not recorded by error.
